# Combined effect of game position and body size on network-based centrality measures performed by young soccer players in small-sided games

**DOI:** 10.3389/fpsyg.2022.873518

**Published:** 2022-08-22

**Authors:** Paulo Henrique Borges, Julio Cesar da Costa, Luiz Fernando Ramos-Silva, Gibson Moreira Praça, Enio Ricardo Vaz Ronque

**Affiliations:** ^1^Department of Physical Education, Center of Sports, Federal University of Santa Catarina, Florianópolis, Brazil; ^2^Department of Physical Education, Center of Physical Education and Sport, State University of Londrina, Londrina, Brazil; ^3^Departamento de Esportes, Universidade Federal de Minas Gerais, Belo Horizonte, Brazil

**Keywords:** soccer (football), growth, body size, performance, youth sports

## Abstract

This study verified the effects of body size and game position on interactions performed by young soccer players in small-sided games (SSG). The sample consisted of 81 Brazilian soccer players (14.4 ± 1.1 years of age). Height, body mass, and trunk-cephalic height were measured. SSG was applied in the GK + 3v3 + GK format, and Social Network Analyses were carried out through filming the games to obtain the following prominence indicators: degree centrality, closeness centrality, degree prestige, and proximity prestige, in addition to network intensity and number of goals scored. Factorial ANCOVA (bone age as covariate) was used to test the effects of game position, body size, and respective interaction on centrality measurements (*p* < 0.05). Similarity between game positions in body size indicators (*p* > 0.05) was observed. The game position affected degree centrality (*p* = 0.01, *η*^2^ = 0.16), closeness centrality (*p*  = 0.01, *η*^2^ = 0.11), and network intensity (*p* = 0.02, *η*^2^ = 0.09), in which midfielders presented the highest network prominence values when compared to defenders and forwards. In conclusion, midfielders are players with high interaction patterns in the main offensive plays, which behavior is independent of body size.

## Introduction

During the first two decades of life, individuals undergo intense biological progress toward maturity. Several morphofunctional changes are observed in this period of life, triggering a gradual increase in anthropometric measures (Figueiredo et al., 2010; Matta et al., 2014) and physical performance in sports tasks (Carvalho et al., 2012; Cunha et al., 2017).

For these reasons, the literature has indicated that young soccer players who reach maturity earlier take advantage in talent-selecting processes (Malina et al., 2000; Coelho-E-Silva et al., 2008), even knowing that the manifestation of specific skills of the modality is weakly related to the body growth process (Malina et al., 2005; Gouvea et al., 2016). This unilateral scenario, biased by the physical dimension, can induce the withdrawal of late players in the maturity process, but with satisfactory tactical-technical qualities, given the impact of the biological maturity state on physical performance.

Adopting a systemic view, capable of verifying the interrelationships of different factors on the tactical-technical dynamics of the match, is recommended to avoid this situation (Williams and Reilly, 2000). Thus, some investigations in the soccer field have brought important contributions to sport professionals and researchers, looking beyond the morphological and functional aspects. It is known that the momentary result of the game (Lago and Martín, 2007), the numerical configuration of the confrontation (Castelão et al., 2014), the training method (Aquino et al., 2015), the number of passes, shots on goal and effectiveness in attack (Hughes and Franks, 2005; Lago-Peñas and Dellal, 2010; Grund, 2012; Kempe et al., 2014; Almeida, 2019), the rapid defensive transition after the loss of the ball (Winter and Pfeiffer, 2016), and the game position (Malta and Travassos, 2014) impact the form soccer players interact and interpret all constraints imposed by teammates and opponents.

Regarding the characteristics of game positions, Rechenchosky et al. (2017) point out that midfielders and forwards frequently make moves to approach and support offensive actions, which is corroborated by Praça et al. (2019a), especially in situations in which teams are winning the game. Furthermore, forwards play at higher intensities and seek physical contact situations (Bloomfield et al., 2007), but midfielders centralize the main offensive actions within the match (Clemente et al., 2015b).

The main role in offensive actions evidenced by midfielders is justified, as these players are placed in regions of the field between defenders and forwards, which involves proximity to the center of the game, aiming at the articulation of the different sectors that make up the team (Sampaio and Maças, 2012). Accordingly, this articulation is expressed by an important engagement in passing distribution in view of the team objectives and offensive strategy (Gonçalves et al., 2014).

These interactions may be understood through the ecological dynamics approach, in which the performance emerges from the co-adaptation between players, who are trying to find the most functional possibility for a specific action (Travassos et al., 2012; Nunes et al., 2020). In this sense, different authors have been investigating the effect of constraints on passing performance in soccer. For example, Grund (2012) observed that the total number of passes performed during the match is related to the number of goals scored, while Clemente et al. (2016a) and Praça et al. (2019b) found that physical performance does not have significant impact on establishing connections.

Although anthropometric characteristics usually have been used to select players rather than playing skills (Deprez et al., 2015), and since young players with larger body proportions and early maturity present advances in the perceptual-cognitive level (Vänttinen et al., 2010; Gonçalves et al., 2020) and better physical performance (Vandendriessche et al., 2012; Teixeira et al., 2018), studies with young soccer players have not yet systematically considered whether the central participation of midfielders in offensive actions is a result only of tasks inherent to the game position (task constraints) or is also influenced by organismic constraints such as body size indicators.

In this context, coaches may take advantage of a deeper understanding of the interplay between game position and organismic constraints with respect to athlete development and game strategy. Considering the information previously available in literature, the initial hypothesis of this study is that midfielders with higher body size indicators show greater centrality in the network. Thus, this study aimed to verify the effects of body size and game position on centrality measures based on the interactions of young soccer players.

## Materials and methods

### Subjects

The sample consisted of 81 young soccer players (14.37 ± 1.12 years; 58.03 ± 10.33 kg; 169.85 ± 10.04 cm) belonging to two soccer teams in Londrina, who played at state level in Brazil. Regarding the weekly training volume, the U-13 category participated on average in 2 weekly training units lasting 120 min each. The U-15 category, in turn, trained on average 5.49 ± 0.49 weekly training units, lasting 180 min each. It was observed that players were familiar with the GK + 3v3 + GK game format, as SSGs are part of the training routine and, therefore, of methodological aspects used by the evaluated clubs, as previously described (Costa et al., 2021).

The following inclusion criteria were adopted: (i) belonging to one of the selected teams; (ii) participating in official competitions for the club; (iii) presenting the free and informed consent form signed by parents or guardians, as well as the assent form. Subjects who presented musculoskeletal injuries during the evaluation period and those who have not completed all project evaluations were excluded. The study was approved by the local ethics committee (Proc. 2650.232/2018).

### Anthropometry and bone age

Body mass was obtained using a digital scale, Seca 813®, with precision of 0.1 kilograms. In turn, height and cephalic trunk height were measured using portable Harpenden® stadiometer and table, with precision of 0.1 cm, following standardization available in literature (Gordon et al., 1988).

Intra-observer reliability was used to analyze the quality of anthropometric measurements. Thus, 16 players were randomly selected 15 days after the first sampling. The following technical measurement errors were identified based on criteria proposed by Perini et al. (2005): body mass: 0.61%, height: 0.72%, and sitting height: 0.62%.

Players were submitted to hand and wrist x-ray at a private clinic. Subsequently, bone age was estimated through the Tanner-Whitehouse 3 method (Tanner et al., 2001), which consists of evaluation of 13 bones of the left hand and wrist according to their development stage.

To assess the intra-observer reliability, 20 x-rays of the hand and wrist were randomly selected. Reproducibility was accomplished using the intraclass correlation coefficient (ICC), observing an ICC value of 0.97 and intra-observer error of 0.26 years.

### Protocol

The use of small-sided games (SSG) has been recommended in literature to carefully evaluate tactical-technical actions that occur within a match (Halouani et al., 2014; Sannicandro and Cofano, 2017), as they simulate the tactical-technical demand and decision-making of the official game (Harrison et al., 2013), in addition to being a tool for identifying/monitoring young talents (Fenner et al., 2016). In this sense, the players were filmed in the GK + 3v3 + GK SSG format. GK + 3v3 + GK was selected as it represents the numerical configuration that captures the core actions of the official game. Thus, the complexity of the formal game was reduced for evaluative purposes while preserving the internal logic of interactions. The spatial dimensions (36 m vs. 27 m) of SSG were derived from the individual playing area of a formal game situation.

The camcorder was positioned high relative to the game plan to capture the entire length of the field, located 6 m above and to one side of the pitch long axis at a distance of 15 m from the pitch. Official soccer rules were adopted, including the offside rule.

**Figure 1:**
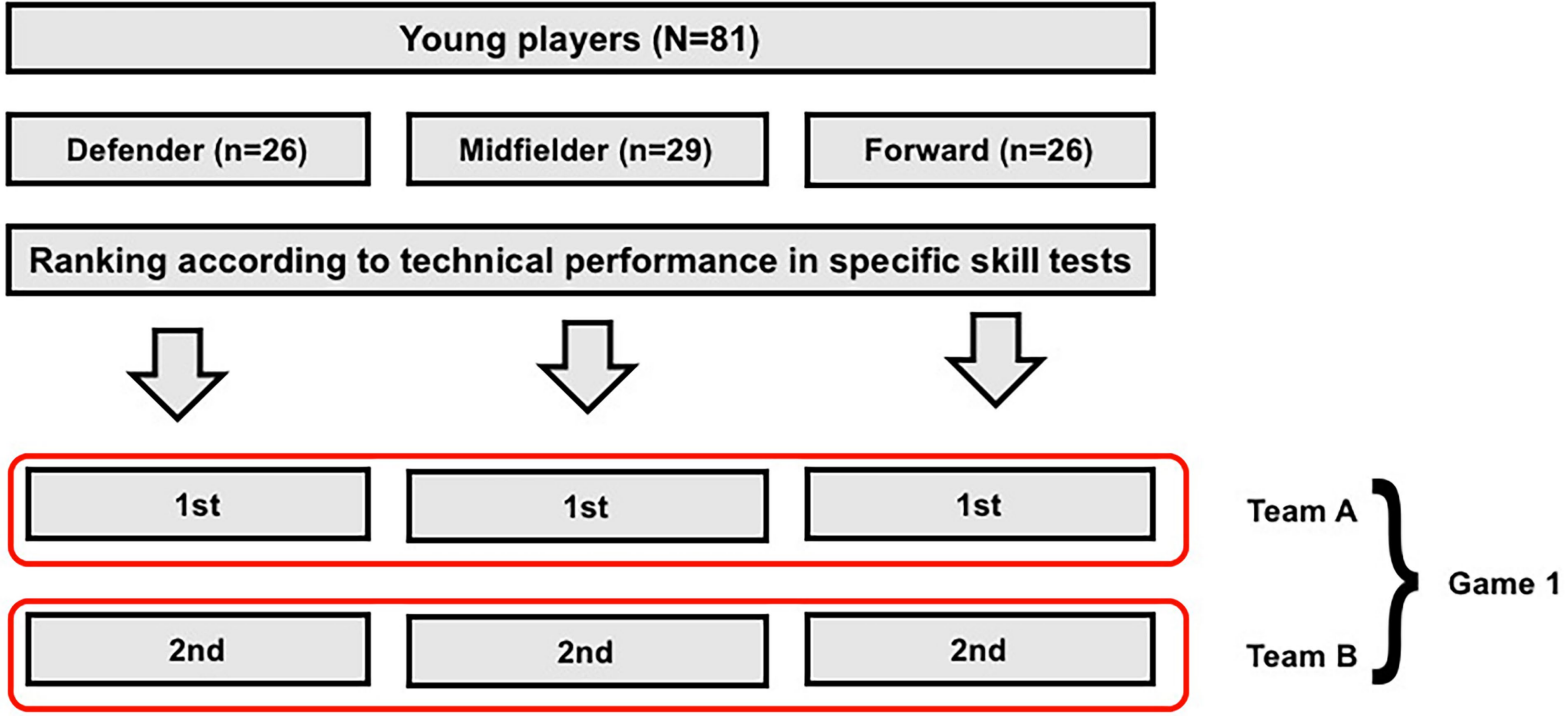
Team composition criteria adopted in this study.

Two tactical-technical criteria were used for the composition of teams: game position and performance in specific skills tests. Thus, teams were formed by a defender (full-back or central defender), a midfielder (defensive midfielder or attacking midfielder), and a forward (striker or winger), according to criteria established in literature (Lemes et al., 2020). The composition of teams was also based on players’ performance on different skills tests: straight-line ball control, zigzag ball control, pass, and kick accuracy tests (Mor and Christian, 1979; Federação Portuguesa de Futebol, 1986). This procedure allowed us achieving a balanced level of performance across teams. In the first confrontation, team A consisted of the best defender, best midfielder and best forward, while team B consisted of the second-best defender, second-best midfielder and second-best forward (Figure 1). This counterbalanced procedure was adopted to allow similar technical performance conditions between teams.

### Social network analysis

**Figure 2:**
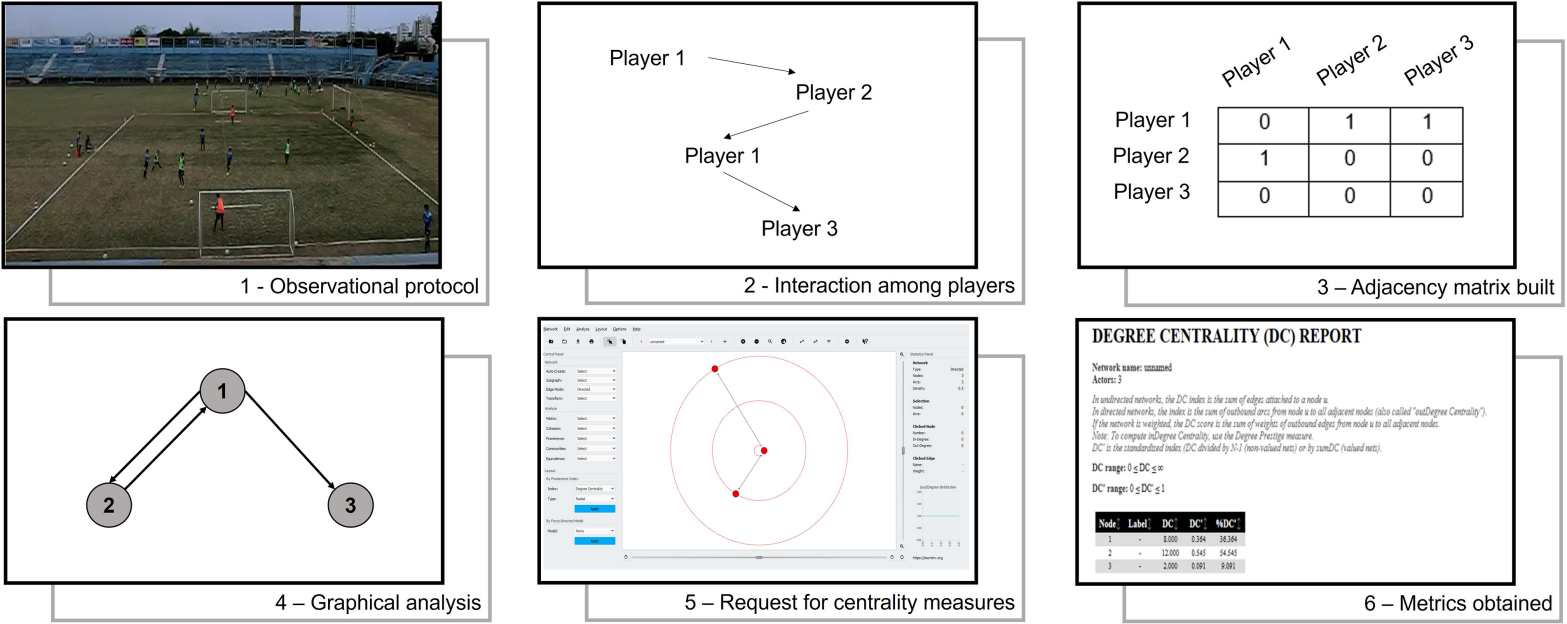
Observational protocol applied in the study.

An observational protocol was used to analyze videos and collect relevant information (Anguera and Hernández-Mendo, 2016). This study is characterized as nomothetic, multidimensional, and punctual (Anguera et al., 2017). The criterion established to mark the interaction between two players was the positive pass (Buldú et al., 2018). The positive pass can be characterized as any technical action in which a player passes the ball to any teammate, who controls the ball and continues playing the game. A situation in which a particular player received the ball and lost it (one-touch) was not considered a positive pass (Figure 2).

Moreover, every positive pass performed to or by the goalkeeper was excluded. Later, adjacency matrices were built for each offensive sequence, and weighted graphs were analyzed (Clemente et al., 2016b).

After this observational analysis, adjacency matrices were inserted into the Social Network Visualizer® software (SocNetV 1.9 (C) 2005–2015 by Dimitris V., Kalamaras). The prominence level of players was measured in social network analysis through the creation and visualization of graphs, with the extraction of the following variables (Frame 1):

**FRAME 1:** Definition and interpretation for team sports about network-based centrality measures.

Variable	Definition	Application for team sports
Degree centrality (DC)	Counts of outbound edges (arcs) from a node to all other nodes connected to it (neighbors)	Indicates the number of links made by the player within the network
Closeness centrality (CC)	Attempts to quantify the importance of actors in terms of their total graph theoretic distance in the social network	Measures the ability of the node (player) to access or send information to other nodes on the network
Degree prestige (DP)	Considers the total number of inbound edges (arcs) from all neighboring nodes connected to it.	Refers to the number of passes (links) that the player receives within the network
Proximity prestige (PP)	Proportion of vertices who can reach a specific node to the average distance the other vertices are from its node	Distance of other teammates from a certain player, suggesting that a player with high proximity prestige values may receive more passes from teammates in the case of a pass.

Clemente et al., 2014, 2016b.

Furthermore, the number of goals scored by each player was identified, as well as the interaction intensity, which refers to the ratio between the degree centrality and the time spent for that execution (Grund, 2012).

Regarding the quality control of observed data, we computed intra- and inter-observer reliability of the network properties in actions performed by 18 young soccer players (22.2% of the total) randomly selected. Intraclass correlation coefficients for intra-observer reliability were: degree centrality (ICC = 0.97), closeness centrality (ICC = 0.89), degree prestige (ICC = 0.99), proximity prestige (ICC = 0.87), and goals scored (ICC = 1.00). Considering inter-observer reliability, the following agreements were obtained: degree centrality (ICC = 0.86), closeness centrality (ICC = 0.82), degree prestige (ICC = 0.96), and goals scored (ICC = 1.00), revealing good reliability (Koo and Li, 2016).

### Statistical analysis

The effects of the game position (independent variable) on body size indicators and experience time (years of systematic practicing) of young soccer players (dependent variables) were tested by the multivariate analysis of variance (MANOVA) after validating the assumptions of data normality and variance–covariance homogeneity, applying the Kolmogorov–Smirnov tests (*p* < 0.05 for all groups) and M of Box for these purposes (Maroco, 2014). Cluster analysis was used from a non-hierarchical k-means procedure to create two body size groups: larger (*n* = 59; 63.22 ± 5.60 kg; 174.70 ± 5.45 cm) and smaller (*n* = 22; 44.10 ± 6.44 kg; 156.84 ± 7.69 cm). The significant variables entered into the model were: height (*F* = 136.12; *p* < 0.01), body mass (*F* = 171.92; *p* < 0.01), and trunk-cephalic height (*F* = 119, 37; *p* < 0.01).

Subsequently, two-way ANCOVA (bone age as covariate) was used, followed by Bonferroni *post hoc*, to test the effects of body size (larger and smaller), game position (defender, midfielder, and forward), and respective interaction between factors on the network-based centrality measures of soccer players. Eta squared (*Ƞ*^2^) was used to analyze the effect size of comparisons. Descriptive and inferential statistical analyses were performed using the SPSS Statistics software (v. 23, IBM SPSS, Chicago, IL), considering 5% significance level.

## Results

Information regarding body size indicators and experience time of young soccer players is described in Table 1. No difference in these characteristics regarding game positions was observed (*F*_mean_ = 1.21; *p* > 0.05).

**Table 1:** Descriptive statistics of body size and experience time indicators of young soccer players as a function of game position (*N* = 81).

	Defender (*n* = 26)	Midfielder (*n* = 29)	Forward (*n* = 26)	*F*	*p*
	Mean (SD)	Mean (SD)	Mean (SD)		
Chronological age (years)	14.26 (1.15)	14.62 (0.95)	14.21 (1.24)	1.11	0.33
Bone age (years)	14.72 (1.40)	14.97 (1.39)	14.30 (1.66)	1.38	0.25
Body mass (kg)	59.66 (10.08)	59.38 (9.53)	54.89 (11.10)	1.80	0.17
Height (cm)	172.33 (10.98)	170.01 (7.53)	167.20 (11.18)	1.72	0.18
Trunk cephalic height (cm)	88.68 (5.96)	88.27 (3.94)	86.50 (7.01)	1.08	0.34
Experience time (years)	6.23 (2.04)	6.62 (2.59)	6.50 (2.30)	0.19	0.82

Additionally, the descriptive statistics related to network-based centrality measures by body size and game position are presented in Table 2. Regarding game positions, midfielders were those with the highest degree centrality (*F*_2,74_ = 7.12; *p* = 0.01), closeness centrality (*F*_2,74_ = 3.92; *p* = 0.01), and network intensity (*F*_2,74_ = 3.86; *p* = 0.02). Interactions between factors “body size” and “game position” were not significant (*p* > 0.05). In other words, being a tall or short midfielder did not influence the network prominence manifested by young soccer players.

**Table 2:** Mean and standard deviation of network properties according to body size and game position, and two-way ANCOVA (controlled for bone age) results to test the effects of body size, game position, and interaction between factors on the network properties performed by young soccer players (*N* = 81).

	Body Size (BS)		Game Position (GP)			Effect BS			Effect GP			BS × GP interaction		
	Smaller (*n* = 22)	Larger (*n* = 59)	Defender (*n* = 26)	Midfielder (*n* = 29)	Forward (*n* = 26)	*F*	*p*	*η* ^2^	*F*	*p*	*η* ^2^	*F*	*p*	*η* ^2^
DC	0.33 (0.09)	0.32 (0.10)	0.29 (0.08)	0.38 (0.09)*	0.29 (0.08)	0.20	0.65	0.03	7.12	0.01	0.16	0.15	0.85	0.01
CC	1.76 (0.77)	1.84 (1.01)	1.59 (0.90)	2.24 (1.05)*	1.58 (0.72)	0.31	0.57	0.01	3.92	0.01	0.11	0.31	0.72	0.01
DP	0.33 (0.10)	0.32 (0.07)	0.31 (0.08)	0.31 (0.05)	0.36 (0.10)	2.53	0.11	0.03	2.84	0.06	0.07	0.90	0.40	0.02
PP	0.27 (013)	0.27 (0.12)	0.29 (0.14)	0.28 (0.13)	0.25 (0.10)	0.04	0.82	0.01	1.32	0.27	0.03	1.71	0.18	0.04
NI	1.01 (0.32)	1.06 (0.47)	0.96 (0.41)	1.23 (0.49)*	0.92 (0.31)	0.49	0.48	0.01	3.86	0.02	0.09	0.28	0.75	0.08
GS	0.77 (0.75)	1.38 (1.30)	1.15 (1.31)	1.17 (0.92)	1.34 (1.38)	0.30	0.58	0.04	0.21	0.80	0.05	0.42	0.65	0.01

DC, degree centrality (%); CC, closeness centrality (d); DP, degree prestige (%); PP, proximity prestige (d); NI, network intensity (passes per minutes); GS, goals scored (quantity).

* Significant difference from midfielders to the other game positions.

## Discussion

This study verified the effects of body size and game position on network-based centrality measures that young soccer players perform in small-sided games. The study’s primary hypothesis was partially confirmed: midfielders presented the most significant prominence in offensive plays. However, the emergence of this tactical-technical action proved to be independent of body size evidenced by young players. Furthermore, although body size indicators are still used as one of the criteria for the selection of talents and composition of a group (Helsen et al., 2005), the number of interactions that emerged in the GK + 3v3 + GK small-sided game are not associated with organismic constraints related to anthropometric measures.

Investigations on anthropometric and maturity characteristics according to different game positions bring controversial results. Deprez et al. (2015) identified a trend in the profile of elite players throughout the sports training process in a study conducted with 744 young soccer players belonging to professional clubs in Belgium: defenders are, on average, taller; midfielders are tougher and better at driving the ball; forwards are smaller, delayed in the maturation process, but faster and more agile. On the other hand, Coelho-E-Silva et al. (2010) evaluated 114 young Portuguese soccer players in the under-14 category and found similar values for indicators of body size, bone age, experience time, physical performance, and specific skills between different game positions, which corroborates the results found in the present study with Brazilian players.

Malina et al. (2009) reported that during adolescence, leg growing processes contribute more than trunk cephalic height to the average height of boys. It could possibly affect all game positions in the same way and not impact interactions performed by players. Then, player characteristics based on game positions are possibly not standardized in soccer since the prospection of athletes and selection of talents are related to the particular strategy of each club and/or federation, which in turn is influenced by cultural, economic, social, and climatic issues (Musch and Hay, 1999).

Additionally, considering that decision-making in team sports consists of a complex process that emerges from several interaction constraints, the identification of prominent players, who substantially contribute to building offensive plays, is important in the context of training young people, since coaches can use this information to manipulate task constraints in order to set boundaries for given action modes to emerge (Clemente et al., 2015a).

In this sense, different studies corroborate the results found in this investigation, highlighting the prominence of midfielders both in the official game (Clemente et al., 2015b, 2016a; Praça et al., 2019a) and in small-sided games (Praça et al., 2017). The region of the field occupied by these players favors the emerging of support movements for the ball carrier to consequently build offensive plays (Gonçalves et al., 2014), which possibly justifies the results found by Sporis et al. (2009) and Bradley et al. (2010), who found higher maximum heart rates and total distances covered by midfielders compared to defenders and forwards.

Furthermore, in practical settings, the reduction in the geodesic distance between midfielders to the other nodes of the network, expressed in this study through the closeness centrality variable, can be understood as an action that helps to solve the problems of the offensive phase of the game, allowing the fulfillment of its operational, tactical principles, i.e., keeping ball possession, progressing with the ball to the opposite goal, and shooting at the goal (Costa et al., 2011a,b). This approach to the ball carrier helps develop an offensive play through the exchange of passes between teammates, which potentially contributes to victory in the respective confrontation (Grund, 2012; Clemente et al., 2015b).

Moreover, body size did not affect the capacity of players to explore passing opportunities with teammates in small-sided games (GK + 3vGK + 3). Therefore, in the scope of training and playing of young U-13 and U-15 soccer players, the development of a central role during the construction of offensive plays is not dependent on body size indicators presented by subjects, even in an invasion sport marked by intense physical contact. This tactical-technical independence is possibly linked to other factors, such as tactical performance, which refers to the selection of appropriate actions within the context of the game (McPherson, 1994).

Collectively, the results found in this study, combined with other previously published investigations, allow us concluding that the adoption of a favorable position for the continuation of the offensive play by reducing the geodesic distance between nodes (players) of the network (team) does not depend on body size. This evidence suggests that the influence of morphofunctional constraints on the central role of midfielders appears to be quite limited, given the inherent complexity of the game (Duarte et al., 2012).

As a study limitation, the number of age groups evaluated stands out, making it impossible to expand the conclusions of this study to other age groups and small-sided game formats. Moreover, the methodological choice of using small-sided games during 2 periods of 4 min each, could make the expansion of these results to official matches difficult.

However, in a sport marked by the selection of physically larger athletes to the detriment of those who are late in this process, the results of this study showed the limits of comprehending the complex process of talent development in soccer using unidimensional criteria. As practical applications of this study, soccer coaches in the U-13 and U-15 categories should avoid unidimensional criteria for talent selection and prospection, selecting young players based on their ability to adapt and make decisions in different scenarios.

Furthermore, going through different game positions from the age of 12 to 15 years using a variety of deliberate play and practice settings, as recommended by the Developmental Model of Sport Participation (Côté et al., 2014; Côté and Vierimaa, 2014) can help soccer players to present different tactical-technical skills about the demands inherent to the different tasks assigned to the team. Professionals involved in training young people can place them in midfield positions to encourage them to learn and offer support to the ball carrier and centralize tactical-technical actions, favoring the emerging of these actions, regardless of body size.

## Data availability statement

The raw data supporting the conclusions of this article will be made available by the authors, without undue reservation.

## Ethical statement

The studies involving human participants were reviewed and approved by Comitê de Ética e Pesquisa da Universidade Estadual de Londrina. Written informed consent to participate in this study was provided by the participants’ legal guardian/next of kin.

## Author contributions

All authors listed have made a substantial, direct, and intellectual contribution to the work and approved it for publication.

## Funding

We gratefully acknowledge funding from the Coordenação de Aperfeiçoamento de Pessoal de Nível Superior - Finance Code 001 (CAPES/BRAZIL). We also acknowledge the Coordenação de Aperfeiçoamento de Pessoal de Nível Superior for a master’s scholarship conceded to JCC (88882.448382/2019-01), and LFRS (88882.448432/2019-01); and The Conselho Nacional de Desenvolvimento Científico e Tecnológico (CNPq/BRAZIL) for the research productivity grant by ERVR (314701/2021-4).

## Conflict of interest

The authors declare that the research was conducted in the absence of any commercial or financial relationships that could be considered as a potential conflict of interest.

## Publisher’s note

All claims expressed in this article are solely those of the authors and do not necessarily represent those of their affiliated organizations, or those of the publisher, the editors and the reviewers. Any product that may be evaluated in this article, or claim that may be made by its manufacturer, is not guaranteed or endorsed by the publisher.

## References

[ref1] Almeida C. H. (2019). Comparison of successful offensive sequences in the group stage of 2018 FIFA world cup: eliminated vs. qualified teams. Sci. Med. Footb. 3, 238–244. doi: 10.1080/24733938.2019.1613557

[ref2] Anguera M. T. Camerino O. Castañer M. Sánchez-Algarra P. Onwuegbuzie A. J. (2017). The specificity of observational studies in physical activity and sports sciences: moving forward in mixed methods research and proposals for achieving quantitative and qualitative symmetry. Front. Psychol. 8:2196. doi: 10.3389/fpsyg.2017.02196, PMID: 29312061 PMC5742273

[ref3] Anguera M. T. Hernández-Mendo A. (2016). Advances in mixed methods observational studies in sports sciences. Cuad. Psic. Dep. 45, 742–755. doi: 10.1080/03055698.2018.1516630

[ref5] Aquino R. L. Q. T. Marques R. F. R. Gonçalves L. G. C. Vieira L. H. P. Bedo B. L. D. S. de Moraes C.. (2015). Proposta de sistematização de ensino do futebol baseada em jogos: Desenvolvimento do conhecimento tático em jogadores com 10 e 11 anos de idade. Motricidade 11, 115–128. doi: 10.6063/motricidade.3724

[ref7] Bloomfield J. Polman R. O’donoghue P. (2007). Physical demands of different positions in FA Premier League soccer. J. Sports Sci. Med. 6, 63–70. PMID: 24149226 PMC3778701

[ref8] Bradley P. S. Mascio M. Peart D. Olsen P. Sheldon B. (2010). High-intensity activity profiles of elite soccer players at different performance levels. J. Strength Cond. Res. 24, 2343–2351. doi: 10.1519/JSC.0B013E3181AEB1B3, PMID: 19918194

[ref9] Buldú J. M. Busquets J. Martínez J. H. Herrera-Diestra J. L. Echegoyen I. Galeano J.. (2018). Using network science to analyse football passing networks: dynamics, space, time, and the multilayer nature of the game. Front. Psychol. 9:1900. doi: 10.3389/fpsyg.2018.01900, PMID: 30349500 PMC6186964

[ref10] Carvalho H. M. Coelho-E-Silva M. Valente-Dos-Santos J. Gonçalves R. S. Philippaerts R. Malina R. (2012). Scaling lower-limb isokinetic strength for biological maturation and body size in adolescent basketball players. Eur. J. Appl. Physiol. 112, 2881–2889. doi: 10.1007/s00421-011-2259-7, PMID: 22138868

[ref11] Castelão D. Garganta J. Santos R. Teoldo I. (2014). Comparison of tactical behaviour and performance of youth soccer players in 3v3 and 5v5 small-sided games. Int. J. Perform. Anal. Sport 14, 801–813. doi: 10.1080/24748668.2014.11868759

[ref200] Clemente F. M. Couceiro M. S. Martins F. M. L. Mendes R. S. (2014). Using network metrics to investigate football team players’ connection: a pilot study. Motriz 20, 262–271. doi: 10.1590/S1980-65742014000300004, PMID: 25964816

[ref12] Clemente F. M. Couceiro M. S. Martins F. M. L. Mendes R. S. (2015a). Using network metrics in soccer: a macro-analysis. J. Hum. Kinet. 45, 123–134. doi: 10.1515/hukin-2015-0013, PMID: 25964816 PMC4415825

[ref13] Clemente F. M. Figueiredo A. J. Martins F. M. L. Mendes R. S. Wong D. P. (2016a). Physical and technical performances are not associated with tactical prominence in U14 soccer matches. Res. Sports Med. 24, 352–362. doi: 10.1080/15438627.2016.1222277 27533018

[ref14] Clemente F. M. Martins F. M. L. Mendes R. S. (2016b). Social Network Analysis Applied to Team Sports Analysis. Cham: Springer International Publishing.

[ref15] Clemente F. M. Martins F. M. L. Wong D. P. Kalamaras D. Mendes R. S. (2015b). Midfielder as the prominent participant in the building attack: A network analysis of national teams in FIFA world cup 2014. Int. J. Perform. Anal. Sport 15, 704–722. doi: 10.1080/24748668.2015.11868825

[ref16] Coelho-E-Silva M. J. Figueiredo A. J. Moreira Carvalho H. Malina R. M. (2008). Functional capacities and sport-specific skills of 14- to 15-year-old male basketball players: size and maturity effects. Eur. J. Sport Sci. 8, 277–285. doi: 10.1080/17461390802117177

[ref17] Coelho-E-Silva M. J. Figueiredo A. J. Simïes F. Seabra A. Natal A. Vaeyens R.. (2010). Discrimination of U-14 soccer players by level and position. Int. J. Sports Med. 31, 790–796. doi: 10.1055/s-0030-1263139, PMID: 20830654

[ref18] Costa J. C. Borges P. H. Ramos-Silva L. F. Weber V. M. R. Moura F. A. Moreira A.. (2021). Do motor performance and specific-skill tests discriminate technical efficiency in small-sided games? Motriz 27:e10210016320. doi: 10.1590/S1980-657420210016320

[ref19] Costa I. T. Garganta J. Greco P. Mesquita I. (2011a). Proposta de avaliação do comportamento tático de jogadores de futebol baseada em principios fundamentais do jogo. Motriz 17, 511–524. doi: 10.1590/S1980-65742011000300014

[ref20] Costa I. T. Garganta J. Greco P. J. Mesquita I. Maia J. (2011b). System of tactical assessment in soccer (FUT-SAT): development and preliminary validation. Motricidade 7, 69–83. doi: 10.6063/motricidade.121

[ref21] Côté J. Turnnidge J. Evans B. (2014). The dynamic process of development through sport. Kinesiol. Slov. 20, 14–26.

[ref22] Côté J. Vierimaa M. (2014). The developmental model of sport participation: 15 years after its first conceptualization. Sci. Sports 29, S63–S69. doi: 10.1016/j.scispo.2014.08.133

[ref23] Cunha G. S. Cumming S. P. Valente-dos-Santos J. Duarte J. P. Silva G. Dourado A. C.. (2017). Interrelationships among jumping power, sprinting power and pubertal status after controlling for size in young male soccer players. Percept. Mot. Skills 124, 329–350. doi: 10.1177/0031512516686720, PMID: 28361651

[ref24] Deprez D. Fransen J. Boone J. Lenoir M. Philippaerts R. Vaeyens R. (2015). Characteristics of high-level youth soccer players: variation by playing position. J. Sports Sci. 33, 243–254. doi: 10.1080/02640414.2014.934707, PMID: 24998472

[ref25] Duarte R. Araujo D. Correia V. Davids K. (2012). Sports teams as superorganisms: implications of sociobiological models of behaviour for research and practice in team sports performance analysis. Sports Med. 42, 633–642. doi: 10.2165/11632450-000000000-00000, PMID: 22715927

[ref26] Federação Portuguesa de Futebol (1986). Habilidades e destrezas do futebol: os skills do futebol. Lisbon: Federação Portuguesa de Futebol.

[ref27] Fenner J. S. Iga J. Unnithan V. (2016). The evaluation of small-sided games as a talent identification tool in highly trained prepubertal soccer players. J. Sport. Sci. 34, 1983–1990. doi: 10.1080/02640414.2016.1149602, PMID: 26939880

[ref28] Figueiredo A. J. Coelho-E-Silva M. J. Cumming S. P. Malina R. M. (2010). Size and maturity mismatch in youth soccer players 11-to 14-years-old. Pediatr. Exerc. Sci. 22, 596–612. doi: 10.1123/pes.22.4.596, PMID: 21242608

[ref29] Gonçalves B. Figueira B. E. Maçãs V. Sampaio J. (2014). Effect of player position on movement behaviour, physical and physiological performances during an 11-a-side football game. J. Sports Sci. 32, 191–199. doi: 10.1080/02640414.2013.816761, PMID: 24016056

[ref30] Gonçalves E. Noce F. Barbosa M. A. M. Figueiredo A. J. Hackfort D. Teoldo I. (2020). Correlation of the peripheral perception with the maturation and the effect of the peripheral perception on the tactical behaviour of soccer players. Int. J. Sport. Exerc. Psychol. 18, 687–699. doi: 10.1080/1612197X.2017.1329222

[ref31] Gordon C. C. Chumlea W. C. Roche A. F. (1988). “Stature, recumbent length, and weight,” in Anthropometric Standardization Reference Manual. eds. Lohman T. G. Roche A. Martorell R. (Champaign: Human Kinetics Books), 3–8.

[ref32] Gouvea M. Cyrino E. S. Ribeiro A. S. da Silva D. R. P. Ohara D. Valente-Dos-Santos J.. (2016). Influence of skeletal maturity on size, function and sport-specific technical skills in youth soccer players. Int. J. Sports Med. 37, 464–469. doi: 10.1055/s-0035-1569370, PMID: 26990721

[ref34] Grund T. U. (2012). Network structure and team performance: The case of English premier league soccer teams. Soc. Netw. 34, 682–690. doi: 10.1016/j.socnet.2012.08.004

[ref35] Halouani J. Chtourou H. Gabbett T. Chaouachi A. Chamari K. (2014). Small-sided games in team sports training: a brief review. J. Strength Cond. Res. 28, 3594–3618. doi: 10.1519/JSC.0000000000000564 24918302

[ref36] Harrison C. B. Nicholas D. G. Kinugasa T. Kilding A. (2013). Quantification of physiological, movement, and technical outputs during a novel small-sided game in young team sport athletes. J. Strength Cond. Res. 27, 2861–2868. doi: 10.1519/JSC.0B013E318280C98D, PMID: 23254547

[ref37] Helsen W. F. van Winckel J. Williams A. M. (2005). The relative age effect in youth soccer across Europe. J. Sports Sci. 23, 629–636. doi: 10.1080/02640410400021310, PMID: 16195011

[ref38] Hughes M. Franks I. (2005). Analysis of passing sequences, shots and goals in soccer. J. Sports Sci. 23, 509–514. doi: 10.1080/02640410410001716779, PMID: 16194998

[ref39] Kempe M. Nopp S. Vogelbein M. Memmert D. (2014). Possession vs. direct play: evaluating tactical behavior in elite soccer. Int. J. Comput. Sci. Sport. 4, 35–41. doi: 10.5923/s.sports.201401.05

[ref40] Koo T. K. Li M. Y. (2016). A guideline of selecting and reporting intraclass correlation coefficients for reliability research. J. Chiropr. Med. 15, 155–163. doi: 10.1016/j.jcm.2016.02.012, PMID: 27330520 PMC4913118

[ref42] Lago C. Martín R. (2007). Determinants of possession of the ball in soccer. J. Sports Sci. 25, 969–974. doi: 10.1080/02640410600944626, PMID: 17497397

[ref43] Lago-Peñas C. Dellal A. (2010). Ball possession strategies in elite soccer according to the evolution of the match-score: the influence of situational variables. J. Hum. Kinet. 25, 93–100. doi: 10.2478/v10078-010-0036-z

[ref44] Lemes J. C. Luchesi M. Diniz L. B. F. Bredt S. D. G. T. Chagas M. H. Praça G. M. (2020). Influence of pitch size and age category on the physical and physiological responses of young football players during small-sided games using GPS devices. Res. Sports Med. 28, 206–216. doi: 10.1080/15438627.2019.1643349, PMID: 31303051

[ref45] Malina R. M. Bouchard C. Bar-Or O. (2009). Growth, Maturation, and Physical Activity. São Paulo: Phorte.

[ref46] Malina R. M. Cumming S. P. Kontos A. P. Eisenmann J. C. Ribeiro B. Aroso J. (2005). Maturity-associated variation in sport-specific skills of youth soccer players aged 13-15 years. J. Sports Sci. 23, 515–522. doi: 10.1080/02640410410001729928, PMID: 16194999

[ref47] Malina R. M. Peñareyes M. E. Eisenmann J. C. Horta L. Rodrigues J. Miller R. (2000). Height, mass and skeletal maturity of elite Portuguese soccer players aged 11–16 years. J. Sports Sci. 18, 685–693. doi: 10.1080/02640410050120069, PMID: 11043894

[ref48] Malta P. Travassos B. (2014). Caraterização da transição defesa-ataque de uma equipa de Futebol. Motricidade 10, 27–37. doi: 10.6063/motricidade.10(1).1544

[ref49] Maroco J. (2014). Análise Estatística com o SPSS Statistics. 6th Edn. Lisbon: Report Number.

[ref50] Matta M. d. O. Figueiredo A. J. B. Garcia E. S. Seabra A. F. T. (2014). Perfil morfológico, maturacional, funcional e técnico de jovens futebolistas Brasileiro. Rev. Bras. Cineantropom. Desempenho Hum. 16, 277–286. doi: 10.5007/1980-0037.2014v16n3p277

[ref51] McPherson S. L. (1994). The development of sport expertise: mapping the tactical domain. Quest 46, 223–240. doi: 10.1080/00336297.1994.10484123

[ref52] Mor D. Christian V. (1979). The development of a skill test battery to measure general soccer ability. N C. J. Health Phys. Educ. 15, 30–39.

[ref53] Musch J. Hay R. (1999). The relative age effect in soccer: cross-cultural evidence for a systematic discrimination against children born late in the competition year. Sociol. Sport J. 16, 54–64. doi: 10.1123/ssj.16.1.54

[ref54] Nunes N. A. Gonçalves B. Davids K. Esteves P. Travassos B. (2020). How manipulation of playing area dimensions in ball possession games constrains physical effort and technical actions in under-11, under-15 and under-23 soccer players. Res. Sports Med. 29, 170–184. doi: 10.1080/15438627.2020.1770760, PMID: 32452730

[ref55] Perini T. A. Lameira De Oliveira G. Dos J. Ornellas S. Palha De Oliveira F. (2005). Cálculo do erro técnico de medição em antropometria. Rev. Bras. Med. Esporte 11, 81–85. doi: 10.1590/S1517-86922005000100009

[ref56] Praça G. M. Clemente F. M. de Andrade A. G. P. Morales J. C. P. Greco P. J. (2017). Network analysis in small-sided and conditioned soccer games: The influence of additional players and playing position. Kinesiology 49, 185–193. doi: 10.26582/k.49.2.8

[ref57] Praça G. M. Lima B. B. Bredt S. d. G. T. Sousa R. B. e. Clemente F. M. Andrade A. G. P. d. (2019a). Influence of match status on players’ prominence and teams’ network properties during 2018 FIFA World Cup. Front. Psychol. 10:695. doi: 10.3389/fpsyg.2019.00695, PMID: 30984084 PMC6447613

[ref58] Praça G. M. Sousa R. B. Greco P. J. (2019b). Influence of aerobic power on youth players’ tactical behavior and network properties during football small-sided games. Sports 7, 1–8. doi: 10.3390/sports7030073, PMID: 30934657 PMC6473423

[ref59] Rechenchosky L. Borges P. H. Menegassi V. M. Jaime M. D. O. Guilherme J. Teoldo I.. (2017). Comparison of tactical principles efficiency among soccer players from different game positions. Hum. Mov. 18, 31–38. doi: 10.1515/humo-2017-0040

[ref60] Sampaio J. Maças V. (2012). Measuring tactical behaviour in football. Int. J. Sports Med. 33, 395–401. doi: 10.1055/S-0031-1301320, PMID: 22377947

[ref61] Sannicandro I. Cofano G. (2017). Small-sided games in young soccer players: physical and technical variables. MOJ Sport Med. 1, 1–4. doi: 10.15406/mojsm.2017.01.00001

[ref62] Sporis G. Jukic I. Ostojic S. M. Milanovic D. (2009). Fitness profiling in soccer: physical and physiologic characteristics of elite players. J. Strength Cond. Res. 23, 1947–1953. doi: 10.1519/jsc.0b013e3181b3e141, PMID: 19704378

[ref63] Tanner J. M. Whitehouse R. H. Cameron N. Marshal W. A. Healy M. J. R. Goldstein N. H. (2001). Assessment of Skeletal Maturity and Prediction of adult Height (TW3 Method). London WB Saunders.

[ref64] Teixeira A. S. Guglielmo L. G. A. Fernandes-da-Silva J. Konarski J. M. Costa D. Duarte J. P.. (2018). Skeletal maturity and oxygen uptake in youth soccer controlling for concurrent size descriptors. PLoS One 13, e0205976. doi: 10.1371/journal.pone.0205976, PMID: 30335836 PMC6193706

[ref65] Travassos B. Araujo D. Davids K. Vilar L. Esteves P. Vanda C. (2012). Informational contraints shape emergent functional behaviours during performance of interceptive action in team sports. Psychol. Sport Exerc. 13, 216–223. doi: 10.1016/j.psychsport.2011.11.009

[ref66] Vandendriessche J. B. Vaeyens R. Vandorpe B. Lenoir M. Lefevre J. Philippaerts R. M. (2012). Biological maturation, morphology, fitness, and motor coordination as part of a selection strategy in the search for international youth soccer players (age 15-16 years). J. Sports Sci. 30, 1695–1703. doi: 10.1080/02640414.2011.652654, PMID: 22296038

[ref67] Vänttinen T. Blomqvist M. Luhtanen P. Häkkinen K. (2010). Effects of age and soccer expertise on general tests of perceptual and motor performance among adolescent soccer players. Percept. Mot. Skills 110, 675–692. doi: 10.2466/pms.110.3.675-692, PMID: 20681323

[ref68] Williams A. M. Reilly T. (2000). Talent identification and development in soccer. J. Sports Sci. 18, 657–667. doi: 10.1080/02640410050120041 11043892

[ref69] Winter C. Pfeiffer M. (2016). Tactical metrics that discriminate winning, drawing and losing teams in UEFA Euro 2012®. J. Sports Sci. 34, 486–492. doi: 10.1080/02640414.2015.1099714, PMID: 26508419

